# Empirical analysis of the International Classification of Functioning, Disability and Health (ICF) using structural equation modeling

**DOI:** 10.1590/bjpt-rbf.2014.0168

**Published:** 2016-06-16

**Authors:** Fabiana C. M. S. Dutra, Marisa C. Mancini, Jorge A. Neves, Renata N. Kirkwood, Rosana F. Sampaio

**Affiliations:** 1 Departamento de Terapia Ocupacional, Instituto de Ciências da Saúde, Universidade Federal do Triângulo Mineiro (UFTM), Uberaba, MG, Brazil; 2 Departamento de Terapia Ocupacional, Programa de Pós-graduação em Ciências da Reabilitação, Universidade Federal de Minas Gerais (UFMG), Belo Horizonte, MG, Brazil; 3 Faculdade de Filosofia e Ciências Humanas, Programa de Pós-graduação em Sociologia, UFMG, Belo Horizonte, MG, Brazil; 4 Programa de Pós-graduação em Ciências da Reabilitação, UFMG, Belo Horizonte, MG, Brazil; 5 Departamento de Fisioterapia, UFMG, Belo Horizonte, MG, Brazil

**Keywords:** ICF, empirical analysis, structural equation model, rehabilitation

## Abstract

**Objective:**

To empirically test the relationships proposed by the International Classification of Functioning, Disability and Health (ICF) among its domains.

**Method:**

The cross-sectional study was completed with 226 adult patients with different health conditions who attended a Brazilian rehabilitation unit. The ICF components were measured with the following instruments: World Health Organization Disability Assessment Instrument II, Functional Independence Measure, Participation Scale, Craig Hospital Inventory of Environmental Factors, and a protocol designed to gather information on body structure and function and personal factors.

**Results:**

Structural equation modeling showed good model adjustment, GFI=0.863; AGFI=0.795; RMSEA=0.028 (90% CI=0.014-0.043). Significant relationships were found between activity and both body structure and function (standard coefficient=0.32; p<0.0001) and participation components (standard coefficient=–0.70; p<0.0001). Environmental and personal factors had a significant effect on the three functioning components (standard coefficient =0.39; p<0.0001; standard coefficient =-0.35; p<0.001, respectively). In contrast, body structure and function had no significant effect on participation (standard coefficient=–0.10; p=0.111) and health conditions had no significant effect on any of the functioning components, i.e., body structure and function, activity, and participation (standard coefficient=–0.12; p=0.128).

**Conclusion:**

Some of the ICF’s proposed relationships across domains were confirmed, while others were not found to be significant. Our results reinforce the contextual dependency of the functioning and disability processes, in addition to putting into perspective the impact of health conditions.

## BULLET POINTS

- Inter-relationships among functioning components had distinct magnitudes.
- Health conditions had no direct effect on functioning and disability.
- Results reinforce the contextual dependency of the functioning process.

## Introduction

Testing models and theories is essential for the validation of conceptual structures that, in turn, allow the description and interpretation of the phenomena represented, such as health/disease, functioning/disability, teaching/learning, and public politics/citizen needs. Models are conceptual formulations that can be thought of as approximations to the studied reality. Models guide clinical reasoning and professional performance and develop a common vocabulary among professionals^1^. Thus, models may support clinical reasoning, decision-making, and advance knowledge.

In 2001, the World Health Organization (WHO) published the International Classification of Functioning, Disability and Health (ICF). The biopsychosocial theory that grounds the ICF proposes that functioning results from the interaction of biological, psychological, environmental, and social factors^2^. This model incorporates information from both individual and social dimensions; it assumes that impairment and disability are not direct consequences of a disease, but are impacted by the physical, political, and social contexts, as illustrated by attitudes towards disability, availability of services, and legislation that ensure the rights of every citizen^2^.

The ICF model has been used in different ways by a number of parties, including researchers, health and rehabilitation clinicians, educators, and legislators. This model has been used, for instance, to analyze the contents of existing clinical instruments as well as provide a conceptual structure to guide the development of new measures to target constructs not yet well explored, such as social participation and the physical and social environments^3^ ^-^ ^9^.

The ICF model proposes three main components to represent functioning: body structure and function (BSF), activity, and participation; further, it states that functioning results from the complex and dynamic interactions among a health condition (HC), personal factors (PF), and environmental factors (EF)^2^. Thus, the model recognizes that functioning and disability are impacted by factors that are both internal and external to the human being. The ICF conceptual structure proposes bidirectional relationships between each component with the nearby construct. This means that impairment of BSF can produce, or be produced by, limitations in activity, which in itself can result in, or be a result of, restriction to participation. All these relationships are influenced by the environmental and personal characteristics surrounding the individual^2^. The extent to which the proposed relationships are generalized to different health conditions and can be observed in various settings helps to validate the conceptual structure of the ICF as well as its applications.

Most studies on the ICF model have focused on its theoretical perspective^10^ ^-^ ^13^, specifically analyzing the philosophical and conceptual foundations of the ICF model. From an empirical perspective, models must be subjected to testing to prove its adequacy and adjustment to real data. To date, studies that provide empirical evidence of the ICF model have targeted specific health conditions or evaluated only specific components of this model, failing to approach its conceptual integrity^7^ ^,^ ^14^ ^-^ ^17^. Recently, the relationships between the components of ICF were tested using measures of self-perception of health^18^. The authors identified direct effects between the components of the model and the perceived health of the participants. Thus, to the best of our knowledge, the empirical evidence on the applicability of the ICF model, using objective measurements and including different health conditions have not yet been explored. Aiming to analyze and empirically test the structure of ICF model, the current study may contribute to its validity and applicability. Specifically, a series of structural equation models was developed to explore the relationships among the domains of the ICF model (impairments in BSF, limitations of activity, and restrictions in participation, PF, and EF) in adult patients attending outpatient public rehabilitation services, with different health conditions.

## Method

This is a cross-sectional study with 226 adult patients of both sexes (age: 18-59 years old) and with different health conditions, selected by convenience. Criteria to participate included ability to walk with or without a walking aid and receiving treatment at the Rehabilitation Reference Center - East Unit in Belo Horizonte, Brazil. Patients who could not understand or execute the tests were excluded.

The Ethics Review Committee of Universidade Federal de Minas Gerais (UFMG), Belo Horizonte, MG, Brazil approved the study protocol (number 132/09), which was conducted in accordance with the Declaration of Helsinki. All participants signed the informed consent form.

Each component of the ICF was assessed independently by selecting its psychometric properties and conceptual coherence with the ICF component^2^. Assessment tools for BSF were identified and selected considering their clinical relevance for rehabilitation practice as well as their inclusion of information from the core sets^19^ ^-^ ^24^. This strategy sought to identify the main difficulties experienced by patients in the BSF domain.

Information on the health condition component was obtained from medical records and classified according to the International Classification of Diseases, 10th version (ICD-10)^25^. Each ICD code was recoded into a binary variable and patients were classified as zero or one, where zero (0) meant the absence of certain diseases and one (1) that the participant had a given pathology. Finally, the various health conditions of the patients were grouped and an index indicating the number of health conditions of each patient was created. In addition to this index, each patient’s ICD was categorized as orthopedic or neurological. In relation to the health status, the stage of the illness was classified as acute or chronic, with the acute condition meaning that the patient was within three months from the onset of the symptoms, illness, or injury.

To inform about the BSF domain, anthropometric measures (height and mass) were taken, followed by testing of muscular strength and flexibility. Pain intensity and submaximal effort were also documented. The Jamar® dynamometer was used to test upper limb strength^26^ and the sit-to-stand test^27^ was used to assess lower limb strength. Upper limb flexibility was assessed with the back scratch test, which measures flexibility during simultaneous performance of shoulder adduction, abduction, internal, and external rotation^27^. Lower limb flexibility was assessed using the fingertip-to-floor test^28^. Pain intensity was measured with the visual analog scale ranging from 0 to 10, where 0 represents the absence of pain and 10 indicates the worst possible pain^29^. The six-minute walking test, considered the best form of assessment of submaximal capacity in patients with different healthy conditions, was also conducted^30^. Body mass index was calculated from each patient’s mass and height obtained during data collection^31^.

The World Health Organization Disability Assessment Schedule II (WHODAS II) and the Functional Independence Measure (FIM) were used to measure the activity component. The WHODAS II informs about disability in six life domains during the previous 30 days^32^. The instrument produces a total score for the corresponding domains of functioning (cognition, mobility, self-care, getting along, life activities, and participation in community activities). The WHODAS II uses a five-point rating scale in which ‘1’ indicates no difficulty and ‘5’ indicates extreme difficulty or inability to perform the activity^32^ ^,^ ^33^.

The FIM measures the patient's level of function and indicates how much assistance is required for the individual to carry out activities of daily living^34^. The scale contains 18 items composed of 13 motor tasks and five cognitive tasks that are rated on a seven point ordinal scale that ranges from total assistance (or complete dependence) to complete independence in basic activities of daily living. The scores range from 18 (lowest) to 126 (highest) indicating level of function^34^.

The Participation Scale (PS) quantifies the restrictions in participation experienced by people affected by different health conditions^5^. This instrument has 18 questions related to the content from the ICF’s Participation component: learning and application of knowledge, communication, mobility, self-care, domestic life, interpersonal interactions and relationships, main areas of life, community, social, and civic life^5^. The total score of the PS can range from zero to 72. The lower the final score, the fewer restrictions the respondent believed were affecting his/her participation^5^.

The Craig Hospital Inventory of Environmental Factors (CHIEF) was used to assess EF. The CHIEF is a questionnaire composed of 25 items, developed to quantify the frequency and the extent to which environmental barriers perceived by the individual affect his or her functioning^3^ ^,^ ^35^. This measure of environmental factors provides three scores (frequency, magnitude, and frequency-magnitude) related to the impact of the environment on functioning. The higher the value of the three scores, the greater the degree to which each element of the physical, social, and political environment contributes to or is perceived as a barrier to the participation of a person with disability^3^.

The selected instruments were standardized, translated and cross-culturally adapted for the Brazilian population and had appropriate psychometric properties, in addition to being grounded on the biopsychosocial model^3^ ^,^ ^5^ ^,^ ^32^ ^-^ ^35^. The appropriateness of these measures has been extensively investigated^24^.

Data on personal factors (PF) were measured with a questionnaire that included information on participant’s sex, age, relationship status, number of children, schooling, occupation, income, and present work situation. The socio-economic index was used to convert the variable “occupation” into “socioeconomic status”^36^. Questions about life habits were gathered to provide information on physical activity levels, smoking habits, and patterns of alcohol intake.

**Figure 1:**
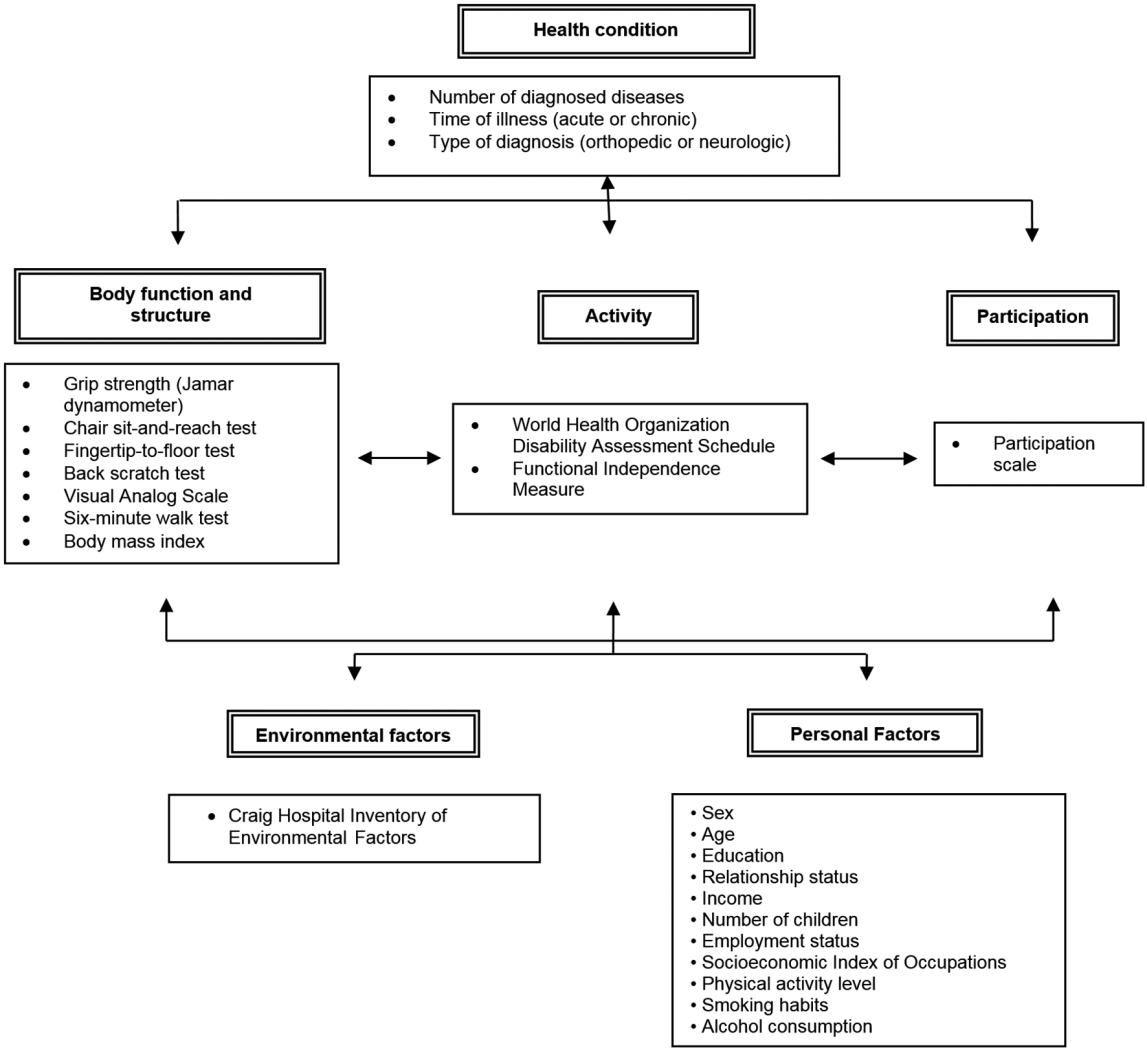
Schema diagram indicating the instrumentation used to measure the different domains of the ICF. Source: Figure adapted from the WHO^2^.

The measurements that addressed each of the ICF domains are shown in Figure 1.

Procedures were performed in five steps: (1) the researcher was trained on the application of all instruments, (2) selection and scheduling of patients who met the inclusion criteria, (3) evaluation of the personal factor domain, (4) evaluation of the activity, participation and environmental factors domains, and (5) evaluation of the BSF domain. The entire evaluation process involving steps 3, 4, and 5 took an average of two hours and was conducted at the Center for Rehabilitation Reference Center - East Unit.

**Figure 2:**
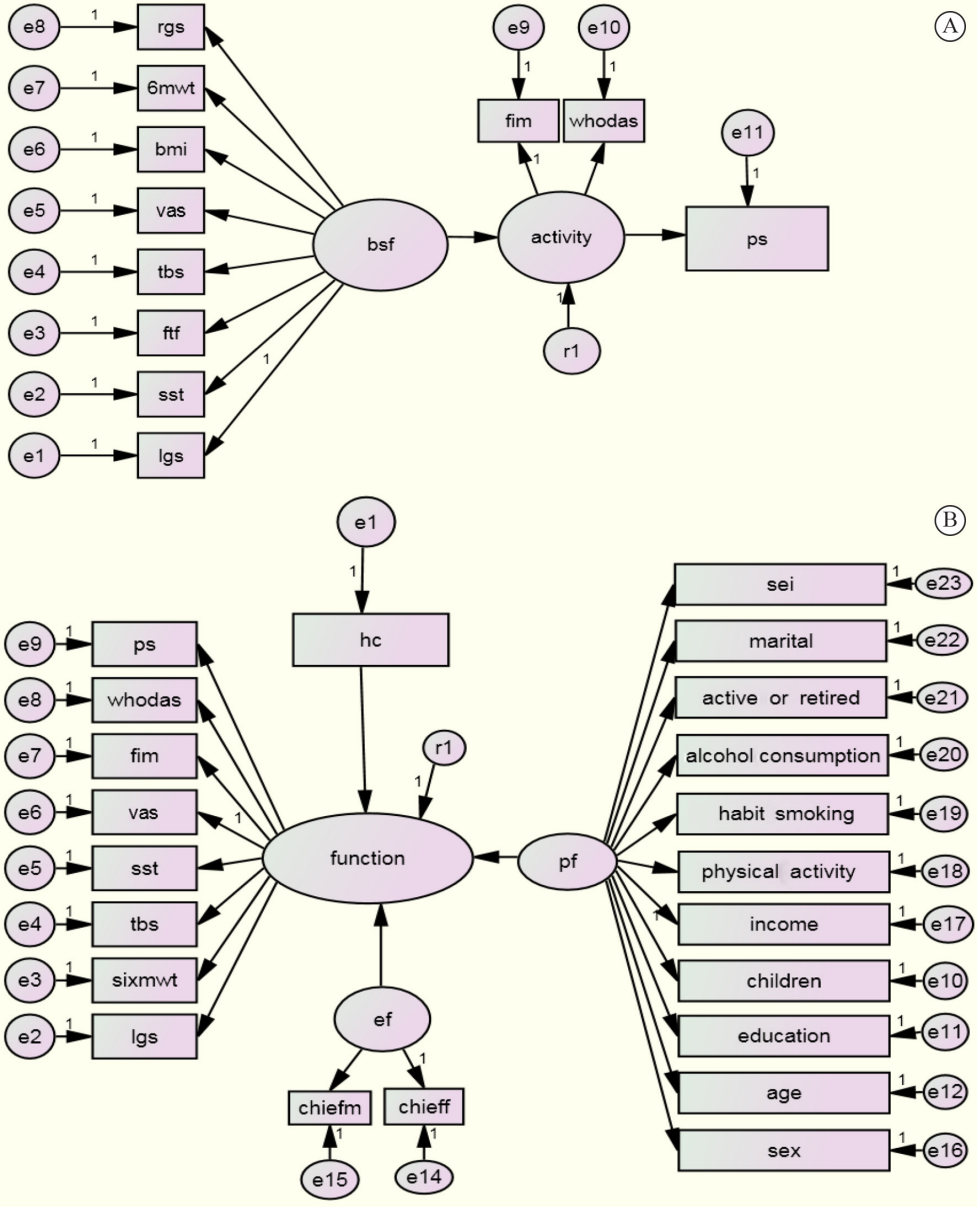
Graphic representation of the structural equation models exploring the relationships among factors representing components of the ICF model. Paths labeled with a “1” were used solely for identification and were therefore not tested. Ovals indicate latent constructs, while rectangles indicate subjacent constructs (i.e., the observed variables). Error values associated with each indicator are shown in small circles labeled with the letter “e”. Standard coefficients are shown above the structural paths between the latent constructs. Standard factorial loads are indicated between the latent constructs and the indicators. Residual errors associated with each latent variable are shown in small circles labeled with the letter “r”. Figure legends: 6MWT: six minute walking test; activeorretired: active at work or retired; age: age in years; alcoholconsumption: regular consumption of alcoholic beverage; vas: visual analog scale; bmi: body mass index; bsf: body structure and function; chieff: frequency of Craig Hospital Inventory of Environmental Factors; chiefm: magnitude of Craig Hospital Inventory of Environmental Factors; children: number of children; education: years of education; ef: environmental factors; function: integration of the components body structure and function, activity, and participation; fim: Functional Independence Measure; ftf: Fingers-to-Floor Test; habitsmoking: smoking; hc: health condition; income: annual income; lgs: left upper limb grip strength; marital: patient is living with partner; pf: personal factors; physicalactivity: regular practice of physical activity; ps: Participation Scale; rgs: right upper limb grip strength; sei: Socioeconomic Index of Occupations; sst: Sit-to-Stand Test; sex: sex of participants; tbs: Back Scratch Test; whodas: World Health Association Disability Assessment Instrument II.

Structural Equation Modeling is a statistical technique that allows the simultaneous modeling of relationships among multiple independent and dependent constructs^37^. This technique is useful for testing explanatory relationships between multiple variables simultaneously, as proposed by the model relationships of the ICF^37^. It combines path analysis, which allows breaking statistical purposes between direct and indirect effects (causal model), and confirmatory factor analysis, which allows the measurement of latent variables (constructs not observed directly) from a set of manifested variables (measurement model), and measures the errors of the observed variables as integrated parts of the model in a single operation^37^. In this study, the first stage of this analysis tested the relationships between the three functioning domains: BSF, activity, and participation (Figure 2A).

These three components were grouped and transformed into a single latent variable named “function”. The other variables (health condition and contextual factors) were further added so the complete ICF conceptual model could be tested (Figure 2B).

To assess the fit of the model the following indices were used: the goodness-of-fit index (GFI), the adjusted goodness-of-fit index (AGFI) indicating the goodness of fit, and the root mean square error of approximation (RMSEA), which reports on the quadratic approximation error. In addition, the 90% confidence intervals (90% CI) were computed.

Based on the existing literature^37^, this study had a sample size appropriate for a solid base for estimation using Structural Equation Modeling. Analyses were performed with the Statistical Package for Social Sciences v.16 (SPSS Inc., Chicago, IL, USA) using the moment structure module. Structural Equation Modeling was developed using the Analysis of Moment Structures v.16 (SPSS Inc. AMOS). The significance level was 0.05.

## Results

The mean age of the participants (n=226) was 42 (SD=12.1) years; 58.0% of them were male, 60.2% lived alone, and the number of children varied from 0-14. The majority of the participants (53.5%) had <8 years of schooling, which corresponds to elementary or middle school education. Only 26.5% of the participants were employed, and the median annual income was US$4,800.00 (range: $0-24,000.00).

The most frequent diagnoses were lower limb fracture, upper limb fracture, stroke, rheumatic diseases, and peripheral nerve injury. Co-morbidities included high blood pressure, dyslipidemia, and type 2 Diabetes. After grouping of diagnoses according to the ICD’s major groups of diseases, their distribution was lesion (40.6%), followed by diseases of the musculoskeletal and connective system (19.4%). For Structural Equation Modeling analysis, diagnoses were divided into two categories: neurological and orthopedic disorders. More than half of the patients (58.4%) had been diagnosed with >1 condition; 58.8% were in the acute phase of the illness process. Most patients were sedentary (75.2%), 38 were smokers (16.8%), and 82 ingested alcohol regularly (36.3%).

The participants’ measures for BSF, activity, participation, and environmental factors are shown in Table 1.

**Table 1:** Measures informing about the ICF components of functioning and disability: body structure and function, activity, participation, and environmental factors (n=226).

Assessments and Tests	Amplitude	Mean (SD)
Body mass index (kg/m^2^)	18.6-40.6	27.1 (5.0)
Right upper limb grip strength (kgf)	0-63.3	28.1 (13.3)
Left upper limb grip strength (kgf)	0-56.0	25.8 (12.4)
Global strength of lower limbs (s)	0-138	23.1 (15.1)
Flexibility of upper limbs (cm)	0-247	20.2 (28.9)
Flexibility of lower limbs (cm)	0-99.0	14.8 (21.0)
Pain intensity	0-10	4.7 (3.1)
Submaximal effort (m)	13-941	430 (140)
WHODAS II*	12-39	23.25 (6.75)
FIM*	79-126	116.7 (7.63)
Participation scale	0-46	17.3 (12.6)
Barrier frequency	0-1.96	0.52 (0.37)
Barrier magnitude	0-1.04	0.36 (0.25)

WHODAS II: World Health Organization Disability Assessment Instrument; FIM: Functional Independence Measure.

### Partial structural model

**Figure 3:**
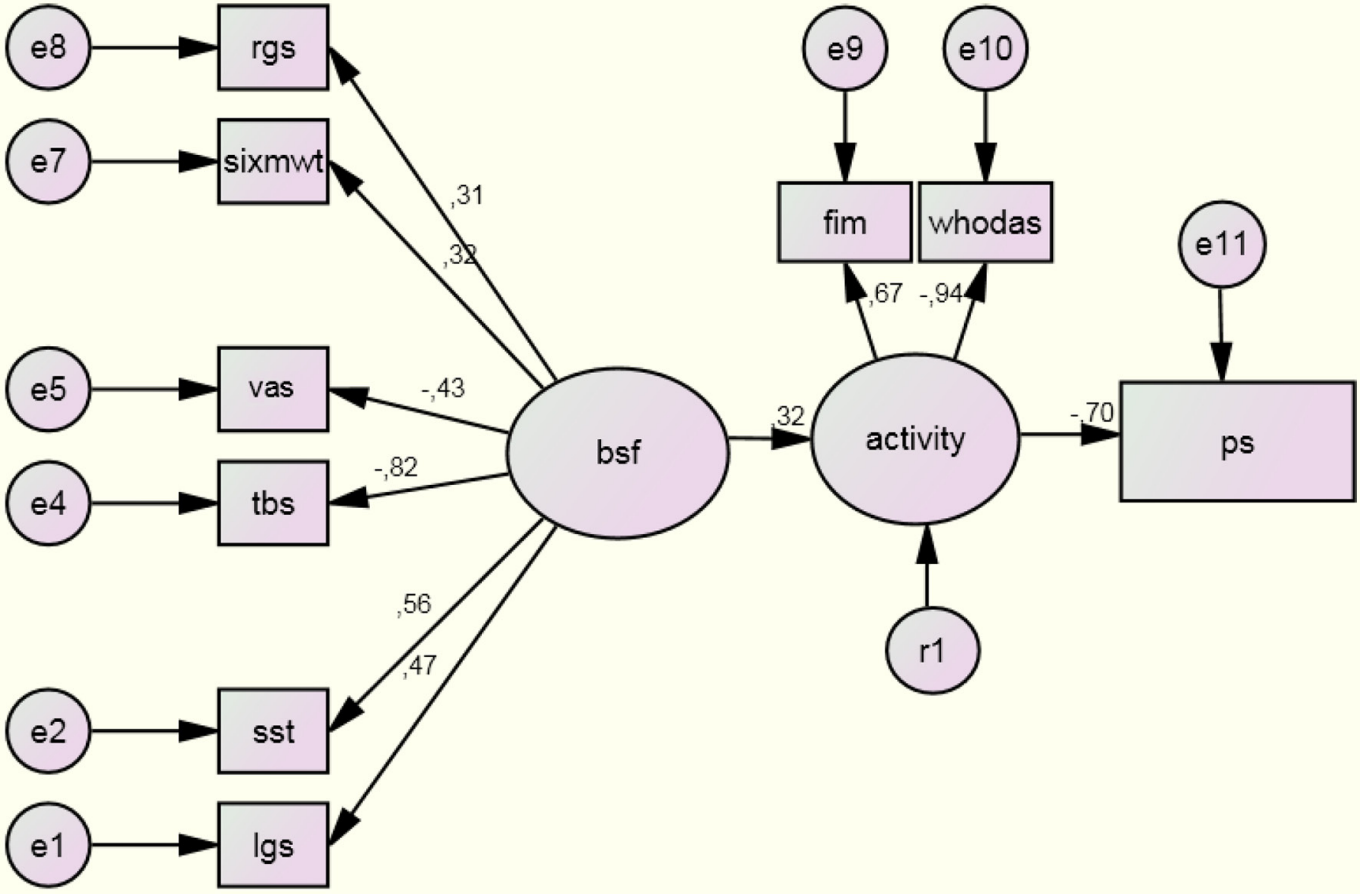
Structural equation model of the relationships between functioning components from the ICF model (n=226; chi-square = 124.1; df=19; GFI=0.886; AGFI=0.781; RMSEA=0.057 [90% CI = 0.031-0.084]). Figure legends: vas: visual analog scale; bsf: body structure and function; fim: Functional Independence Measure; lgs: left upper limb grip strength; ps: Participation Scale; sixmwt: six minute walking test; sst: Sit-to-Stand Test; tbs: Back Scratch Test; WHODAS: World Health Association Disability Assessment Instrument II. The decimal numbers on the arrows indicate the significant correlation coefficients from the Structural Equation Modeling, showing positive and negative (-) associations.

Our first model explored the paths between BSF and activity, activity and participation, and BSF and participation (Figure 2A). We found significant relationships between activity and both BSF (standard coefficient=0.32; p<0.0001) and participation (standard coefficient=–0.70; p<0.0001). The relationship between BSF and participation was not significant (standard coefficient=–0.10; p=0.111), and values <0.3 were obtained for the factor loadings of the fingers-to-floor test, body mass index, and right upper limb grip strength. These were excluded from the model and a new model omitting each of these relationships was built^37^. Generally, the adjustment measures of this model were moderate to good, chi square=124.1; df=19; GFI=0.886; AGFI=0.781; RMSEA=0.057 (CI90%=0.031-0.084). All estimated paths and standardized coefficients of the partial model are shown in Figure 3.

### Complete structural model

**Figure 4:**
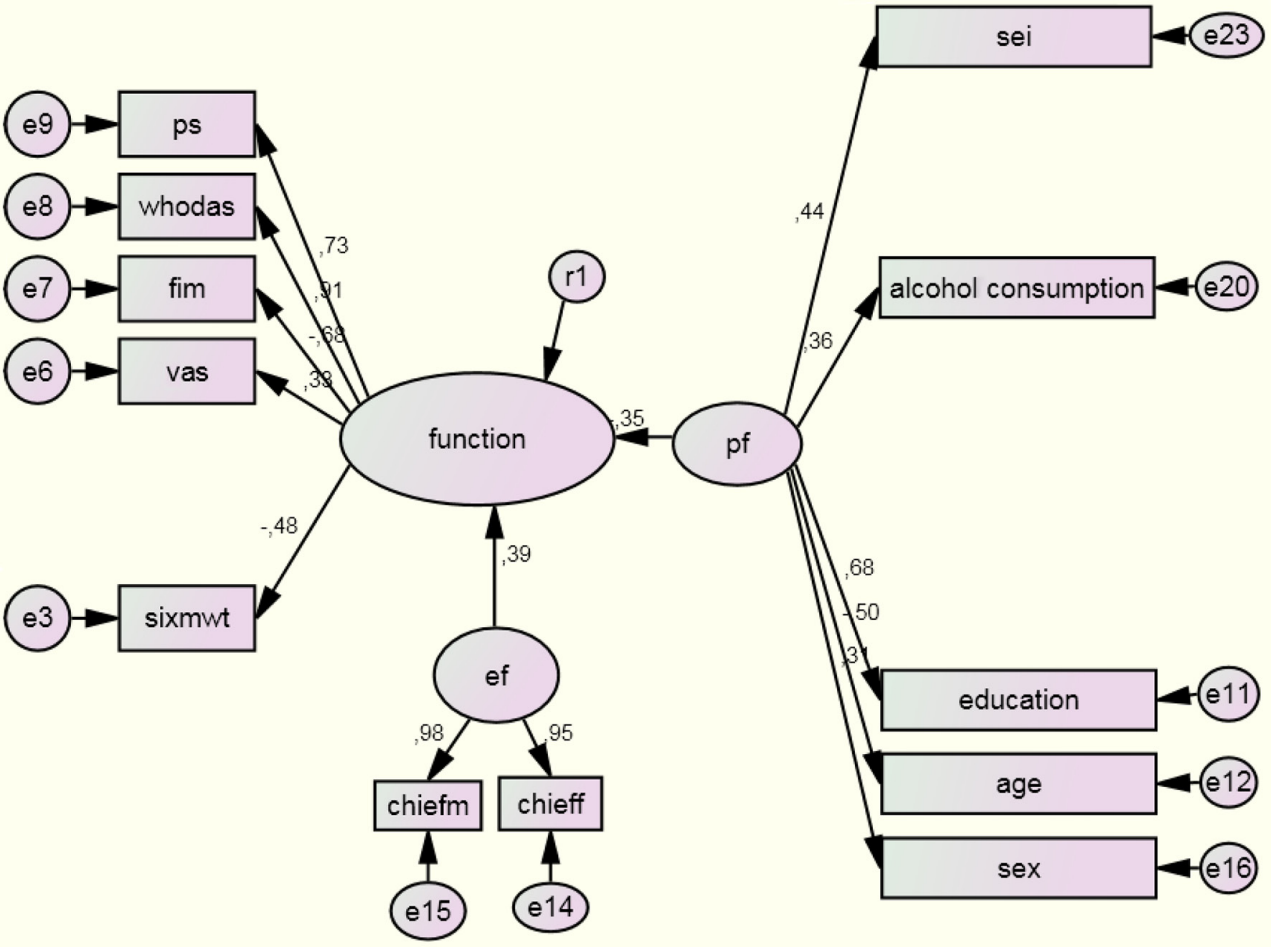
Results of the structural equation model of the complete model of the ICF (n=226; chi square = 252.9; df = 52; GFI=0.863; AGFI=0.795; RMSEA=0.028 [90% CI=0.014-0.043]). Figure legends: age: age in years; alcoholconsumption: regular consumption of alcoholic beverage; vas: visual analog scale; chieff: frequency of Craig Hospital Inventory of Environmental Factors; chiefm: magnitude of Craig Hospital Inventory of Environmental Factors; ef: environmental factors; education: years of education; function: integration of the components body structure and function, activity, and participation; fim: Functional Independence Measure; pf: personal factors; ps: Participation Scale; sei: Socioeconomic Index of Occupations; sex: sex of participants; sixmwt: six minute walking test; whodas: World Health Association Disability Assessment Instrument II. The decimal numbers on the arrows indicate the significant correlation coefficients from the Structural Equation Modeling, showing positive and negative (-) associations.

To test the ICF model, the EF, PF, and HC domains were added to the partial model previously described, allowing the exploration of the paths between “function” (BSF, activity, and participation) and HC, EF, and PF (Figure 2B). In the complete model, the relationship between HC and function was not significant (standard coefficient =–0.12; p=0.128). Likewise, the subjacent construct of the BSF “sit-to-stand test” and subjacent constructs of the PF’s “physical activity”, “smoking habit”, “employment status”, and “relationship status” were also not significant. Left upper limb grip strength, back scratch test, and number of children obtained factor loadings values <0.3, and according to the criteria presented in the literature^37^, these were excluded from the model. A second model excluding each of these non-significant variables was created. The direct effect of contextual factors on the three components of functioning was significant (standard coefficient=0.37; p<0.0001; standard coefficient =–0.34; p<0.001, respectively). Adjustment indices associated with this final model ranged from moderate to good, chi square=252.9; df=52; GFI=0.863; AGFI=0.795; RMSEA=0.028 (CI90%=0.014-0.043). All estimated paths and standardized coefficients of this complete model are shown in Figure 4.

## Discussion

The present study empirically tested the relationships proposed by the ICF conceptual model in patients with different health and stage conditions using Structural Equation Modeling. The results partially support some of the relationships in the ICF model, highlighting the essential influence of the contextual factors on the functioning components.

Our partial model revealed significant relationships between BSF and activity (standard coefficient=32) and between activity and participation (standard coefficient=–70). Further, we found that the magnitude of the influence of BSF on activity was lower than that of the effect of activity on participation. This result highlights the involvement of complex factors to explain participation, not just biological or intrinsic body structures and functions. Although restrictions in participation have been correlated with limitations in activity, the existing evidence suggests that impairment of BSF has shown weak association with, or only indirect effects on, participation, indicating that participation restrictions are not completely explained by impairment of BSF^38^ ^-^ ^42^. Thus, we suggest that rehabilitation professionals cautiously analyze the interventions directed exclusively towards BSF and try to introduce to the therapeutic process actions directed towards other functioning components.

The analysis of the complete ICF model revealed significant relationships between EF and functioning (standard coefficient=0.39) and between PF and functioning components (standard coefficient=–0.35). These results confirmed that both EF and PF influence functioning. Thus, the ICF model shows, in its conceptual structure, the characteristics of a context-dependent phenomenon. It advocates that functioning is influenced by personal characteristics and environmental factors, which represent aspects that are internal and external to the individual. While the WHO recognizes the importance of contextual factors (personal and environmental) on functioning, only in recent years has there been an increase in the number of studies that analyzed the impact of these factors together. The limited information available on the relationship between environmental factors and personal factors and functioning can be justified by the fact that the discussions on these inter-relationships are relatively new. Silva et al.^43^ found that personal factors such as years of schooling and being active in the labor market are conditions that enhance social participation of the patients in rehabilitation. In contrast, the greater the frequency of environmental barriers - especially barriers related to services and assistance, attitudes and support, and physical structure, when analyzed simultaneously with personal factors, increase restrictions on social participation of patients with various diseases/health conditions^43^.

Regarding PF, our results suggest that higher socioeconomic levels, education, alcohol consumption, younger age, and sex (male) are characteristics that have a positive effect on the patients' functioning. Regarding education and occupational status, more years of education and higher occupational status might increase the individual’s access to information, health services, infrastructure, and social support^44^ ^,^ ^45^. Studies analyzing the weekly consumption of alcoholic beverages identified better health perceptions, greater social participation, and lower prevalence of disability in people with moderate consumption of alcohol^43^ ^,^ ^46^ ^-^ ^48^. According to the WHO^49^, mortality and functional limitations among individuals with conditions associated with alcohol consumption outweigh those whose conditions are associated with smoking. It is estimated that, worldwide, alcohol is related to 3.2% of all deaths and 4.0% of disability-adjusted life years (DALY). In developing countries with low mortality rates, such as Brazil, alcohol intake is a risk factor that contributes to the burden of disease, accounting for 6.2% of DALYs^49^. It is also important to consider the effect of the patients’ age and gender on their functioning. Our results indicate that older people and women tend to have more functional limitations compared to younger people and men, respectively. This evidence is similar to that found in other studies^44^ ^,^ ^48^.

The increase in the frequency and magnitude of the barriers present in the daily life of individuals could explain the impact of environmental factors on functioning and disability. When environmental factors are expressed as barriers, evidence indicates a negative impact, leading to disability. However, the availability and access to health services and rehabilitation, as well as environmental facilitators such as support from family and friends, lead to human functioning and help patients adapt to their HC and meet new challenges in life^3^ ^,^ ^42^ ^,^ ^43^ ^,^ ^50^. Thus, disability and functioning are phenomena mediated by the environment, and in order to account fully for the ICF model, it is necessary to incorporate information about both the social and natural environments^12^.

Our results suggest that HC has no direct effect on the functioning components of the ICF model. This result confirms an important conceptual advance of the ICF model for the analysis of functioning. This principle is based on the biopsychosocial perspective that supports the ICF model starting from a multifactorial and complex understanding of disability focused on the interrelationship between biological, social, and personal factors, in addition to the naturalistic understanding of disability focused only on the nosological status of the individual. Indeed, Perenboom et al.^18^ identified a high degree of relationship between the components of health condition and BSF domains and participation. However, it is important to stress that all measures used by Perenboom et al.^18^ were measures of perception. Thus, the association found may be explained by the high correlation between the perceived health of the participants and the perception of wellbeing in the components of BSF and participation.

In our study, HC was operationalized as the number of health conditions, classified by disease groups and time of illness (acute or chronic). However, our results demonstrate that, when analyzing the functioning process from a multidimensional and objective structure, different factors interacted with each other (e.g., biological, individual and social) and the direct effect of HC is no longer significant. It is suggested that, in order to understand the phenomenon of functioning, multiple levels of analysis are needed^2^ ^,^ ^12^.

### Study limitations

This study, as far as we know, is the first to include the objective measurement of all components of the ICF, including analysis of environmental factors. Some of its limitations included some descriptive characteristics of the sample. It was composed of participants with low education (<8 years), who were out of work, with an annual income lower than $ 4,800.00, and most of them with acute conditions. These characteristics illustrate a group with certain specificities and may limit the generalization of our findings. Nevertheless, the influence of such socio-demographic characteristics needs to be examined. Further empirical testing of the WHO model should be conducted with patients of different ages as well as children and youth, providing evidence for the recently developed ICF-CY (for children and youth).

## Conclusions

We confirmed relationships between the BSF and activity, as well as activity and participation. Likewise, we observed an effect of PF and EF on functioning. However, we failed to find evidence supporting an effect of BSF on participation, which suggests that the relationship between these two factors may be mediated by activity.
